# Sub-sector, property rights system, and performance of China’s tourism enterprises: DEA and Luenberger approach

**DOI:** 10.1371/journal.pone.0320928

**Published:** 2025-04-03

**Authors:** Liyang Yang, Nan Zhu, Huiru Bai

**Affiliations:** 1 School of Tourism and Culture Industry, Chengdu University, Chengdu, Sichuan, China; 2 Western Business School, Southwestern University of Finance and Economics, Chengdu, Sichuan, China; University of Naples Federico II Department of Business Economics: Universita degli Studi di Napoli Federico II Dipartimento di Economia Management e Istituzioni, ITALY

## Abstract

This study is designed to delve into the efficiency and total factor productivity (TFP) of tourism enterprises, aiming to uncover the sources of variation in these metrics. To meet this objective, we have employed the data envelopment analysis (DEA) approach and the Luenberger index—making it the inaugural application within the tourism industry—to assess efficiency and TFP. Our findings indicate that the tourism industry boasts high efficiency, with progress in TFP and its component factors. In our quest to identify the determinants of efficiency and TFP, the research has taken into account sub-sectors and the property rights system. It has been revealed that there are notable efficiency disparities among different sub-sectors, while differences in TFP are not significant. When examining the property rights system, we found significant efficiency and TFP differences between state-owned enterprises (SOEs) and non-SOEs. To the best of our knowledge, this research pioneers the application of nonparametric analysis to differentiate the performance disparities in tourism enterprises attributable to the property rights system. Furthermore, we have constructed a decision-making matrix that incorporates an enterprise’s efficiency, TFP, and scale. This matrix allows each enterprise to precisely pinpoint its position, enabling managers to formulate targeted strategies for performance enhancement.

## 1. Introduction

With the complete removal of travel and contact consumption restrictions, there has been a surge in consumer demand, leading to the swift emergence of a new cultural industry. Tourism stands out as a key driver of economic development [1], as it generates economic value by capitalizing on cultural and touristic assets, financial resources, land, and human resources [2]. Moreover, it serves as a connector for various sectors, such as transportation, travel agencies, and hotels, through its extensive industrial chain [3]. Tourism enterprises are the building blocks of the industry, and accurately assessing their performance can provide a reflection of the industry’s overall performance. This is not only advantageous for improving operations and management but also for fostering industrial clustering. However, the tourism industry is highly competitive [4], and the financial performance of enterprises within it is a crucial factor in enhancing their competitiveness. From this perspective, the importance of evaluating the financial performance of tourism enterprises has become increasingly evident.

Tourism businesses are often split into different sub-sectors, like hotels [5–8], travel agencies [9,10], restaurants [11], and maritime tourism [12,13]. A significant feature of today’s tourism enterprises is their involvement in multiple sectors simultaneously, and a key strategy for tourism enterprises to increase their competitive edge is to expand their range of operations and provide better services [14]. For example, hotels might offer not just provide accommodation but also dining and travel advisory services, effectively taking on the roles of travel agencies and restaurants. Obviously, there is tough competition among these various sectors, and it doesn’t make sense to assess an enterprise’s competitiveness within one sub-sector alone. So, when looking at how well tourism enterprises are doing, we need to treat businesses in each sub-sector as similar units for a fair evaluation.

Chinese listed tourism enterprises are governed by two distinct property rights systems: SOEs and non-SOEs. The contentious issue of how property rights systems impact financial performance is under scrutiny, particularly in assessing whether state ownership confers an advantage. A significant divergence exists between SOEs and non-SOEs, especially with respect to privileges, such as the mechanisms for receiving policy incentives, bank credit, and fiscal subsidies [15], and managerial styles, including agency problems [16]. The manner and cost of resource acquisition can benefit the financial efficiency of SOEs; however, there is a tendency among SOE managers to prioritize personal gain over enhancing the efficiency of the enterprises they manage [17]. Given the intertwined development of tourism enterprises with resource allocation and management styles, it is imperative to evaluate the financial performance of these enterprises under varying property rights systems. This assessment is crucial for the industry’s evolution.

The objective of this research is to assess and differentiate the financial performance of tourism enterprises. To be more precise, the study has three main goals: First, to determine the efficiency and TFP of tourism enterprises; second, to explore the variances in efficiency and TFP across different sub-sectors and under various property rights systems; and third, to analyze the characteristics of tourism enterprises and categorize them into distinct types. To achieve these objectives, we employ DEA to calculate the efficiency of the tourism enterprises, and the Luenberger index to measure their TFP. Subsequently, we utilize two nonparametric analysis approach—the Mann-Whitney U and Kruskal-Wallis tests—to identify differences in efficiency and TFP among sub-sectors and property rights systems. Furthermore, the enterprises are profiled based on their efficiency, TFP, and scale, and are classified into eight distinct types based on these attributes.

This study makes several scholarly contributions, which are delineated below. Firstly, we employ the negative DEA and Luenberger index to ascertain efficiency and TFP. Unlike previous DEA models in the literature, which lack the capability to handle negative data, our approach addresses this limitation, thereby, mitigating potential biases in practical applications. And the Luenberger index is based on the directional distance function and is particularly advantageous due to its capacity to concurrently effect a reduction in inputs and an expansion in outputs, a feature not commonly found in traditional indices [18,19]. While the majority of precedent literature has opted for the Malmquist index in TFP calculations, our approach offers a novel perspective. To our knowledge, this is the inaugural study to utilize the Luenberger index to investigate the TFP of tourism enterprises. Secondly, our research extends beyond a general examination of the tourism industry to its sub-sectors. This holistic view contrasts with the prevailing body of work that tends to concentrate on enterprises within a single sector. Thirdly, to our best knowledge, this investigation pioneers the application of nonparametric analysis to elucidate financial performance disparities attributable to varying property rights systems within tourism enterprises. Unlike previous methodologies that have relied heavily on regression analysis, which necessitate presuppositions that may not always be tenable, our nonparametric approach circumvents such prerequisites, offering a more flexible analytical framework. Lastly, we introduce a decision-making matrix that profiles tourism enterprises based on efficiency, TFP, and scale dimensions that encapsulate the multifaceted nature of financial performance. To the best of our knowledge, this study represents the inaugural effort to integrate these three critical elements within a single analytical framework, encapsulating the multifaceted aspects of financial performance. While prior research has often overlooked scale as a performance metric, we argue that scale, as a reflection of long-term, fixed investments, is a pivotal determinant of superior operational performance [20]. This comprehensive approach offers a more nuanced understanding of the financial performance within the tourism industry and establishes the decision-making matrix as a fundamental tool for assessing the competitive strategies of tourism enterprises.

The subsequent sections of this study are methodically organized as delineated herein: Section 2 encompasses a thorough review of the pertinent extant literature, providing a foundational context for our research endeavors. Section 3 elaborates on the methodological framework underpinning our analysis, which includes the deployment of DEA, the Luenberger index, the Mann-Whitney U test, and the Kruskal-Wallis test. Section 4 presents the dataset utilized in this study, detailing the sources and the rationale for its selection. Section 5 is dedicated to an exposition of the findings, offering insights drawn from the empirical analysis. Concluding the discourse, Section 6 offers a synthesis of the study’s contributions and implications.

## 2. Literature review

### 2.1. Performance assessment of tourism enterprise

Enterprise financial performance is one of the most popular concepts in organizational research [21]. Historically, financial performance has been gauged through a spectrum of financial metrics, notably return on assets (ROA) [2,22], return on equity (ROE) [22,23], and stock returns [22,24]. It is axiomatically assumed that enterprises exhibiting superior financial ratios manifest enhanced performance. However, given that individual financial ratios provide only a fragmented assessment of organizational performance [25], there is a pressing need for a holistic performance evaluation framework that integrates multiple financial metrics. The discordance among financial ratios often obscures comparative performance analyses. The efficiency-based approach transcends the limitations inherent in ratio-based measurements and has emerged as a prevalent empirical framework for performance evaluation [26]. Among the efficiency methodologies, DEA and the stochastic frontier approach (SFA) have garnered substantial traction in performance assessments. DEA, a nonparametric technique, eschews the specification of a functional form, thereby circumventing restrictive data constraints. This method necessitates no presupposed functional relationship between inputs and outputs, allowing for diverse physical or monetary units of inputs and outputs without prior knowledge. Moreover, DEA is not stringent in its requirement for large datasets [27]. The efficiency score, derived from the ratio of multiple inputs to outputs, ensures the comparability of performance metrics. In contrast to other efficiency analysis tools, DEA is distinguished by its capacity to furnish actionable improvement insights for inefficient units. Its ability to provide benchmarking information constitutes one of its principal advantages over alternative efficiency methodologies [28]. DEA has been successfully deployed to measure the efficiency of organizations within the service industry [9] and has been extensively applied in the study of tourism enterprise performance. Morey and Dittman [29] were pioneers in employing DEA to assess the efficiency of U.S. tourism enterprises from a managerial performance standpoint. Subsequent research on the performance of tourism companies has proliferated. Geng et al. [30] conducted an analysis of the sustainability performance of tourism enterprises in China, uncovering an improvement in average technical efficiency, albeit with significant fluctuations in operational performance. Kamel et al. [31] evaluated the efficiency of 16 Egyptian tourism enterprises utilizing Window DEA and slack-based measure (SBM) DEA, revealing a marked deterioration in efficiency during the COVID-19 pandemic. It is important to note that the DEA methodologies utilized in the aforementioned literature are not equipped to handle negative data. In instances of financial loss, the profit, a pivotal indicator of financial performance, is often substituted with transformed data, potentially leading to biased results. To facilitate an objective performance evaluation, there is an urgent need to integrate a negative DEA model into performance assessments.

Productivity within the tourism industry is garnering increased scholarly attention as a pivotal indicator of performance. Barros [32] has emphasized the feasibility of quantifying enterprise productivity, highlighting its significant in the tourism industry. Poretti and Heo [33] have gone further to suggest that productivity is a more robust predictor of enterprise value within the tourism industry than traditional profitability metrics. The measurement of productivity is frequently facilitated by two predominant approaches: the Malmquist index [34] and Luenberger index. Existing literature on TFP of tourism enterprises is always based on the Malmquist index [5,35]. However, the Malmquist index is founded on the Shepard distance function, which does not accommodate the simultaneous consideration of input reduction and output expansion, necessitating proportional changes in each variable. In contrast, the Luenberger index addresses these limitations by permitting the concurrent consideration of reducing inputs and increasing outputs, without the requirement for proportional changes in each variable. Furthermore, the Malmquist index presents a limitation in that one must opt for either an output-oriented or an input-oriented perspective, whereas the Luenberger index eschews this requirement and aligns with the economic imperative of profit maximizations for enterprises [36,37]. This methodology has been applied to assess the productivity of organizations in various sectors, including banking [38], education [39], and transportation [40]. However, despite the extensive application of the Malmquist index, there is dearth of literature employing the Luenberger index to measure productivity within the tourism enterprises.

While the performance of tourism enterprises has garnered considerable attention, the extant literature has often focused on single sectors, neglecting the nuanced dynamics of sub-sectors. For instance, studies by Barros and Dieke [27], Aissa and Goaied [41], and Yin et al. [42] have concentrated exclusively on hotel enterprises. Similarly, Köksal and Aksu [9] and Fuentes [10] have based their analyses solely on travel agencies. Karakistsiou et al. [11] have limited their scope to restaurants, and Santos et al. [12] and Chrysafis et al. [13] have utilized data predominantly from nautical tourism. However, the landscape of modern tourism enterprises is characterized by diversified development. Concentrating solely on the internal competitiveness within one sub-sectors is insufficient to address the intensifying competition within the broader tourism industry. This study, therefore, undertakes a comprehensive evaluation of enterprise performance across the entire tourism industry.

### 2.2. Property rights systems and enterprise performance

The discourse on the nexus between property rights system and enterprise performance often invokes rigorous methodological approaches. The Tobit regression model [43] and the bootstrapping procedure [44] are frequently deployed analytical tools. However, it is imperative to recognize that these regression techniques necessitate certain variable prerequisites, such as linearity, the absence of multicollinearity, and normality; a failure to adhere to these conditions may compromise the integrity of the regression findings. Yet, due to objective constraints, including limitations in sample size, the majority of performance research data on tourism enterprises often fall short of meeting the prerequisites of these methodologies. Concurrently, there is a conspicuous deficiency in existing research when it comes to conducting tests for linearity, normality, and collinearity when employing Tobit regression method or bootstrap process, which further amplifies the likelihood of biased outcomes. When data deviates from normality, integrating non-parametric analysis with performance evaluation emerges as a viable strategy. This approach circumvents the stringent variable requirements and aligns seamlessly with the DEA methodological framework. The Mann-Whiteney U and Kruskal-Wallis tests are particularly prominent in the evaluative arsenal of performance assessment [20,28,45]. These methodologies are particularly adept at handling data that exhibit non-normal characteristics, thereby providing a more accurate and nuanced understanding of the performance metrics within the tourism enterprises under review. Despite their utility, prior research has often neglected to leverage nonparametric tests to discern the impact of property rights systems on enterprise performance, an oversight that warrants rectification in future scholarly endeavors.

### 2.3. Performance evaluation and enterprise decision-making

The delineation of the relative positioning of enterprises is instrumental in the formulation of strategic management directives. Scholars such as Hwang and Chang [5] and Yu and Chen [46] have constructed decision-making matrices that encapsulate the efficiency along the horizontal axis and the technological gap along the vertical axis. These matrices facilitate the categorization of hotels into distinct types, each of which can inform tailored managerial strategies. However, the existing literature has often focused solely on efficiency and the change in efficiency, a decomposed indicator of TFP, neglecting the scale of enterprises. It is imperative to recognize that scale is an additional critical factor in performance evaluation, and enterprises of varying scales may necessitate divergent management strategies. The small-scale enterprises, with their agility and cost flexibility, possess distinct advantages [47], whereas their larger counterparts may leverage capital investments for competitive edge [48]. Consequently, the incorporation of scale into decision-making matrices is essential for enterprises in the development of comprehensive management strategies.

## 3. Methodology

### 3.1. DEA method

DEA is a well-established nonparametric methodology for assessing the relative efficiency and productivity of decision-making units (DMUs). Originally conceptualized within the realm of physics, the economic interpretation of efficiency pertains to the optimal allocation of inputs to produce the maximum outputs. In the context of DEA, a DMU is deemed efficient if it can generate a greater quantity of output with a given set of inputs, or equivalently, if it uses fewer inputs to produce the same level of outputs. Traditional efficiency methods, while widely used, have notable limitations, particularly in their inability to rank DMUs that are efficient. However, the super-efficiency DEA model [49] has extended the efficiency ranking to include these efficient units, thereby providing a more nuanced understanding of relative efficiency. This approach utilizes linear programming models with explicit coefficients [8], which facilitates straightforward and interpretable results. Following this advancement, many researchers have focused on constructing and refining super-efficiency models [50–52]. Moreover, traditional DEA models, including those mentioned above, are not designed to handle negative values in input-output indicators, which can be common in practical applications. For instance, when profit is used as an output indicator despite a part of enterprises operating at a loss, traditional models fail to provide accurate efficiency assessments. Addressing this challenge is crucial for enhancing the practical relevance of DEA models. When negative indicators are part of the output, it is essential to employ a super-efficiency model that can accommodate these negative indicators without distorting the efficiency scores of the units under review. The modified slacks-based super-efficiency measure (modified super SBM model) [53] has been instrumental in this regard, enabling the calculation of efficiencies for listed tourism enterprises by incorporating both positive and negative output indicators. In applying the DEA model, it is assumed that there exists a set of n DMUs, each utilizing m inputs to yield s outputs. For each, represents the ith input, and denotes its rth output. The modified super SBM model can be expressed as:


min1+∑i∈Iuiwi−Pi−1−∑r∈Ovrwr+Pr+s.t.xik≥∑j=1,j≠knxijλj−wi−,i∈Iyrk≤∑j=1,j≠knyrjλj+wr+,r∈O∑j=1,j≠knλj=1,λj≥0,j=1,2,...,n,j≠kwr+≤Pr+,r∈Owr+,wi−≥0,∀r∈O,i∈I (1)

where $P_{i}^{-}=\underset{j}{\max}\left\{x_{ij}\right\}-\underset{j}{\min}\left\{x_{ij}\right\},i=1,2,...,m,P_{r}^{+}=\underset{j}{\max}\left\{y_{rj}\right\}-\underset{j}{\min}\left\{y_{rj}\right\},r=1,2,...,s,$

$I= \{\left.i\right|\left.P_{i}^{-}>0,i=1,2,...,m\right\},O= \{\left.r\right|\left.P_{r}^{+}>0,r=1,2,...,s\right\}$, $\lambda_{j}$ represents the density vector, while $w_{i}^{-}$ and $w_{r}^{+}$ denote the super-efficiency slacks, signifying potential input savings and output surpluses, respectively. The weights $u_{i}$ and $v_{r}$ satisfy $\sum_{i=1}^{m}u_{i}=1$ and $\sum_{r=1}^{s}v_{r}=1$. Model (1) is formulated to determine the super-efficiency of by minimizing the slacks $w_{i}^{-}$ and $w_{r}^{+}$, thereby yielding an optimal value that is at least one, indicating efficiency. For inefficient DMUs, the following improved SBM model (2) is provided:


min1−∑i∈Iuisi−Pi−1+∑r∈Ovrsr+Pr+s.t.xik=∑j=1,j≠knxijλj−wi−*+si−,i∈Iyrk=∑j=1,j≠knyrjλj+wr+*−sr+,r∈O∑j=1,j≠knλj=1,λj≥0,j=1,2,...,n,j≠ksi−,sr+≥0,∀i∈I,r∈O (2)

where $s_{i}^{-}$ and $s_{r}^{+}$ are designated as inefficient slacks, representing, respectively, the input excesses and output shortfalls.

Utilizing the aforementioned pair of models, the efficiency of DMUs can be assessed through a two-tiered process: initially, Model (1) is implemented to discern the efficiency of the DMUs; subsequently, in the second stage, Model (2) is executed to refine this assessment. Thereby, the efficiency of the specific unit is delineated by Equation (3):


θk*=1+∑i∈Iuiwik−*Pi−1−∑r∈Ovrwrk+*Pr+,if1+∑i∈Iuiwik−*Pi−1−∑r∈Ovrwrk+*Pr+>11−∑i∈Iuisik−*Pi−1+∑r∈Ovrsik−*Pr+,else (3)

The efficiency outcome is divided by one, where a value exceeding one signifies an efficient DMU, whereas a value below one indicates an inefficient DMU.

The Luenberger index is invoked to compute TFP, as delineated in Equation (4):


$$TFP_{k}=\frac{1}{2}\left\{\left[D^{t+1}\left(x_{k}^{t+1},y_{k}^{t+1}\right)-D^{t+1}\left(x_{k}^{t},y_{k}^{t}\right)\right]+\left[D^{t}\left(x_{k}^{t+1},y_{k}^{t+1}\right)-D^{t}\left(x_{k}^{t},y_{k}^{t}\right)\right]\right\}$$ (4)

where $D^{q}\left(x_{k}^{p},y_{k}^{p}\right)$ signifies the distance to the production frontier. Utilizing the $L_{1}$ distance to represent $D^{q}\left(x_{k}^{p},y_{k}^{p}\right)$, the distance is articulated in Equation (5):


$$D^{q}\left(x_{k}^{p},y_{k}^{p}\right)= \{\begin{array}{l} \sum_{i\in I}\frac{w_{ik}^{-*p}}{P_{i}^{-p}}+\sum_{r\in O}\frac{w_{rk}^{+*p}}{P_{r}^{+p}},if\left(w_{ik}^{-*p}+w_{rk}^{+*p}\right)>0\\ -\left(\sum_{i\in I}\frac{s_{ik}^{-*p}}{P_{i}^{-p}}+\sum_{r\in O}\frac{s_{rk}^{+*p}}{P_{r}^{+p}}\right),else \end{array}$$ (5)

with p and q denoting temporal benchmarks, specifically, p, q = t, t + 1.

A positive value of indicates progress in TFP; conversely, a negative value suggests TFP regress. The TFP index can be further decomposed into efficiency change (EC) and technical change (TC), as formulated in Equations (6) and (7), respectively, while adhering to the constraint presented in Equation (8).


$$EC_{k}=D^{t+1}\left(x_{k}^{t+1},y_{k}^{t+1}\right)-D^{t}\left(x_{k}^{t},y_{k}^{t}\right)$$ (6)

$$TC_{k}=\frac{1}{2}\left\{\left[D^{t}\left(x_{k}^{t+1},y_{k}^{t+1}\right)-D^{t+1}\left(x_{k}^{t+1},y_{k}^{t+1}\right)\right]+\left[D^{t}\left(x_{k}^{t},y_{k}^{t}\right)-D^{t+1}\left(x_{k}^{t},y_{k}^{t}\right)\right]\right\}$$ (7)

$$TFP_{k}=EC_{k}+TC_{k}$$ (8)

EC mirrors the optimization of internal resource utilization and factor allocation within a single enterprise. A positive EC value signifies an improvement in efficiency, denoting progress in the organization’s operational effectiveness. Conversely, a negative EC value indicates a decline in efficiency, reflecting a regression in the enterprise’s ability to optimally deploy its resources. TC represents the incorporation of novel technological insights and innovation in the realm of product services. A positive TC value points to advancements in technology, suggesting that innovations have enhanced the production process or the quality of output. On the other hand, a negative TC value implies a lack of technological advancement or even a backsliding in the adoption of efficient production techniques.

### 3.2. Mann-Whitney U and Kruskal-Wallis Test

The Mann-Whitney U and Kruskal-Wallis tests are utilized to ascertain the efficiency and TFP disparities among samples. The Mann-Whitney U test serves as the nonparametric counterpart to the independent t-test, facilitating the comparison of two independent samples that do not adhere to a normal distribution. The underlying principle of this methodology posits that if two groups are derived from the same distribution and are randomly assigned labels, the values of the two distinct groups should exhibit a roughly equivalent distribution. The Mann-Whitney U test evaluates all possible rankings between the data points, with the null and alternative hypotheses formulated as follows:

- H_0_: There is no difference in the data from the two groups.
- H_1_: There is a difference in the data between the two groups.

The procedural steps for conducting the Mann-Whitney U test are delineated as follows:

- Step 1: Combine the data from both groups into a single set, sort them in ascending order, and assign ranks to the data points.
- Step 2: Calculate the sum of ranks for each group, denoting $R_{1}$ as the sum of ranks for group one and $R_{2}$ as the sum for group two.
- Step 3: Determine the U statistic for each group using the formulas $U_{1}=R_{1}-\frac{n_{1}\left(n_{1}+1\right)}{2}$ and $U_{2}=R_{2}-\frac{n_{2}\left(n_{2}+1\right)}{2}$, where $n_{1}$ and $n_{2}$ represent the sample sizes of groups 1 and 2, respectively. The test statistic U is the lower value between $U_{1}$ and $U_{2}$.
- Step 4: Employ the test statistic to decide whether to reject or fail to reject the null hypothesis based on the chosen significance level α and the critical value, or P-value, obtained from the Mann-Whitney U distribution table. If the calculated P-value is less than α, the null hypothesis is rejected; otherwise, it is not rejected.

The Kruskal-Wallis test expands upon the Mann-Whitney U test to accommodate three or more independent groups. It is recognized as the nonparametric alternative to one-way ANOVA when the assumption of normality is not met. The null and alternative hypotheses are stated as:

H_0_: There is no difference in the data from the groups.
H_1_: There is a difference in the data from the groups.

The fundamental steps for conducting the Kruskal-Wallis test are as follows:

Step 1: Combine the data from all groups into a single set, sort them in ascending order, and assign ranks to the data points.
Step 2: Sum the ranks of each group.
Step 3: Compute the H statistic using the formula $H=\left[\frac{12}{n\left(n+1\right)}\sum_{i=1}^{k}\frac{T_{j}^{2}}{n_{j}}\right]-3\left(n+1\right)$, where n is the total number of observations across all groups, k is the number of groups, $T_{j}$ is the sum of ranks in the $j^{th}$ group, and $n_{j}$ is the size of the $j^{th}$ group.
Step 4: Ascertain the critical value, or P-value, based on the calculated H statistic, and decide whether to reject or fail to reject the null hypothesis based on the significance level α and the P-value. If the calculated P-value is less than α, the null hypothesis is rejected; otherwise, it is not rejected.

## 4. Materials

Sample Selection Methodology. Corporations typically derive profits through the production and sale of goods, which generate revenue. However, the tourism industry, classified as a service sector, primarily offers intangible products in the form of various services. Unlike tangible goods, services are characterized by their non-storability, perishability, and heterogeneity. Given that these attributes are ubiquitous across tourism products, the tourism enterprises under scrutiny in this study can be considered homogeneous entities. Nevertheless, the extensive range of industries encompassed by listed tourism enterprises poses challenges in defining clear boundaries, leading to inconsistencies in sample selection across existing literature. To address this, the current study filters a sample of 29 listed tourism enterprises from the period 2013 to 2022, utilizing the industry classification standards established by the China Securities Regulatory Commission. The selection process excludes enterprises designated as ST (Special Treatment) and * ST. The selected enterprises are categorized into four distinct sub-sectors: travel, hotels, restaurant, and entertainment facilities, in accordance with the aforementioned classification criteria. The resultant dataset is an unbalanced panel dataset, thereby mitigating the potential for survivor bias, as references in literature [54].

Variable Selection. Tourism enterprises are labor-intensive, profit-oriented organizations that generate revenue and realize profits through the strategic allocation of limited costs and human resources. These enterprises capitalize on the efficient utilization for their workforce to optimize the financial outcomes, highlighting the significance of human capital in their operational and financial success. Consequently, the evaluate the efficiency and TFP of these enterprises, the analysis is anchored in two key input indicators and two output indicators.

The inputs are delineated by two indicators:

(1) Operating costs: This variable encompasses all resources procured in the provision of services. Similar variables have been utilized as input indicators in the literature, as referenced in [35,41,55,56].
(2) Employees: This is quantified by the number of full-time equivalent employees, which is crucial for enhancing enterprise competitiveness, as highlighted in [57]. This metric serves as an input indicator in several studies, including [9,10,55].

The outputs are delineated by two additional indicators:

(1) Annual turnover: This is aligned with the methodology of previous literature [7,41,55,58–60], as it signifies a measure of the enterprises’ achievement of their objectives.
(2) Profits after tax: Employed as an output indicator in [1], this is defined as the net earnings of a business after accounting for all taxation-related expenses. It serves as an assessment of the actual earnings of a business and its operational viability.

The data for these variables are sourced from the annual reports of each enterprise. Table 1 presents the descriptive statistics for the dataset.

**Table 1:** The statistics of inputs and outputs.

	Indicator	Min	Max	Mean	SD
Inputs	Operating costs (billion)	0.016	44.799	2.091	5.422
	Employees (people)	348	40801	4474.942	6826.577
Outputs	Annual turnover(billion)	0.047	66.941	3.339	8.237
	Profits after tax (billion)	-1.767	12.365	0.254	1.122

^a^Min- minimize, Max- maximize, Mean- average, SD- standard deviation.

Table 2 delineates the correlation coefficients between the input and output variables. A statistically significant positive correlation is discerned between each input and each output variable, suggesting that an increment in input is associated with a corresponding increase in output. This finding aligns with the hypothesis of constant returns to scale, which is a foundational assumption in DEA methodology.

**Table 2:** Correlation coefficients among inputs and outputs.

Correlation	Operating costs	Employees	Annual turnover	Profits after tax
Operating costs	1			
Employees	0.381**	1		
Annual turnover	0.966**	0.522**	1	
Profits after tax	0.863**	0.342**	0.906**	1

a ** Correlation is significant at the 0.01 level.

## 5. Results

### 5.1. Industry level

Table 3 illustrates the efficiency outcomes for the enterprises across the period from 2013 to 2022.

**Table 3:** Efficiency and effective enterprise from 2013 to 2022.

Year	Efficiency	Effective enterprise	
		Number	%
2013	0.911	8	36.36%
2014	0.913	8	33.33%
2015	0.912	9	37.50%
2016	0.938	8	33.33%
2017	0.961	10	38.46%
2018	0.941	8	29.63%
2019	0.926	7	25.93%
2020	0.940	8	28.57%
2021	0.947	7	24.14%
2022	0.958	9	31.03%
Mean	0.936		

**Fig 1:**
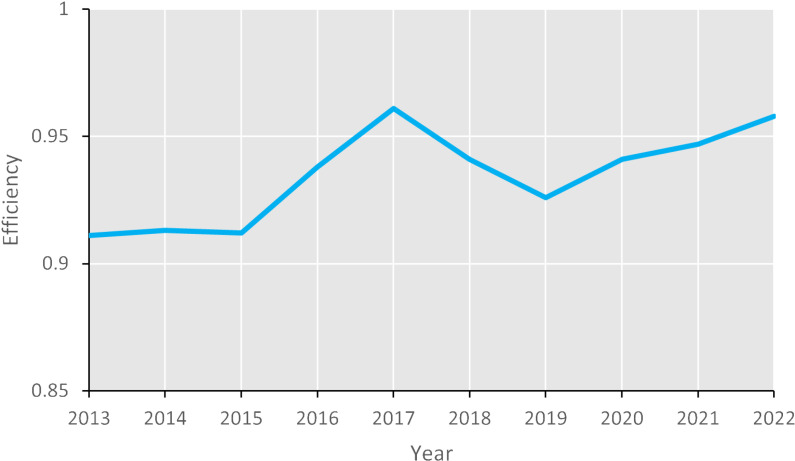
Industry’s efficiency from 2013 to 2022.

The overall efficiency of the tourism industry is reflected in the sample year’s efficiency score of 0.936 for the tourism enterprises, which is comparatively elevated. Nonetheless, the presence of a 6.4% inefficiency quotient suggests that there is substantial scope for enhancement. Thisimplies that, on average, the industry could further optimize its operation by reducing both operating costs and the scale of its workforce, while concurrently striving to augment annual turnover and profits after tax. The efficiency trajectory of the tourism enterprises from 2013 to 2022 exhibits a fluctuating pattern, as depicted in Fig 1. The efficiency was relatively stable between 2013 and 2015, followed by periods of rapid ascent and subsequent decline. It reached its zenith at 0.961 in 2017, only to recede to 0.926 in 2019. The pronounced increase elucidates the rationale behind tourism’s status as the world’s largest and most rapidly expanding sector [61]. Conversely, the downturn in 2019 underscores the devastating impact of the COVID-19 pandemic on the tourism industry [62]. Post-2019, there was a resurgence in efficiency, albeit gradual, attributable to the slow relaxation of travel restrictions and lingering apprehensions regarding mass infections from virus mutations [63]. This uptick also underscores the tourism industry’s remarkable resilience and adaptability in the face of catastrophic or unforeseen events [64]. Table 3 provides further insights, indicating that each year, approximately one-third of the enterprises demonstrated efficient operations. The peak efficiency was attained in 2017, which recorded the highest proportion of efficiency enterprises. Notably, in that year, the ten efficiently operating enterprises accounted for 38.46% of the total, substantially enhancing the overall efficiency of the industry.

The Kolmogorov-Smirnov and Shapiro-Wilk tests have been applied to determine the normality of the efficiencies, with the results depicted in Table 4.

**Table 4:** The normality of efficiency.

	Kolmogorov-Smirnow			Shapiro-Wilk		
	Statistic	df	Sig.	Statistic	df	Sig.
Efficiency	0.425	252	0.000	0.214	252	0.000

It can be posited that P-value approaching zero indicates significant deviation of the efficiencies from a normal distribution, thereby fulfilling the preconditions for the application of the Mann-Whiteney U and Kruskal-Wallis tests.

Table 5 delineates the efficiency scores and the outcomes of the Kruskal-Wallis test across various sub-sectors. A P-value approaching zero signifies a highly significant disparity in efficiency levels among the sub-sectors at the 99% confidence level. The data indicate that the entertainment facilities sector achieved the highest efficiency rating of 0.981 during the sample year, outperforming both the travel and hotel sectors. In contrast, the restaurant sector registered the lowest efficiency score of 0.899, which corresponds to an operational inefficiency of 10.1%, suggesting a substantial scope for enhancement.

**Table 5:** The efficiency and slacks of sub-sectors.

Year	Travel	Hotel	Restaurant	Entertainment facility
Efficiency				
2013	0.919	0.886	0.867	0.945
2014	0.930	0.873	0.821	0.952
2015	0.919	0.913	0.803	0.963
2016	0.942	0.936	0.878	0.962
2017	0.957	0.974	0.931	0.985
2018	0.932	0.951	0.908	0.989
2019	0.911	0.952	0.897	0.982
2020	0.961	0.783	0.963	0.995
2021	0.967	0.844	0.938	0.994
2022	0.963	0.933	0.925	0.994
Mean	0.941	0.904	0.899	0.981
Kruskal-Wallis Test	22.913			
Degree of freedom	3			
Sig	0.000			
Slacks				
Operating costs (billion)	0.147	0.131	0.004	0.00
Employees (people)	1293.404	5038.226	2862.907	425.171
Annual turnover (billion)	0.507	0.157	0.300	0.153
Profits after tax (billion)	0.456	0.589	0.383	0.189

**Fig 2:**
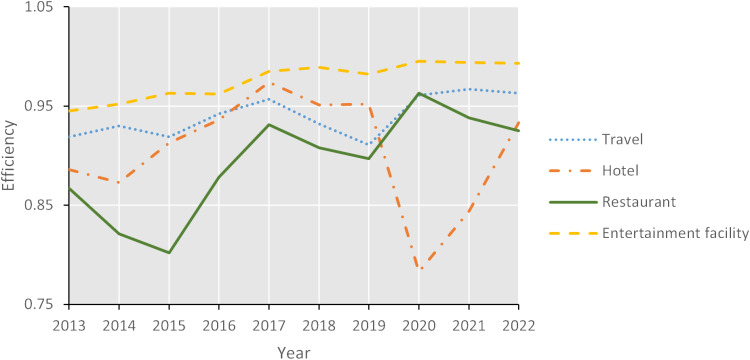
Sub-sectors’ efficiency from 2013 to 2022.

The disparities in efficiency are further elucidated through an analysis of slacks. Relative to other sub-sectors, the entertainment facilities sector exhibited the minimal excess in inputs and shortfalls in outputs. In contrast, the restaurant sectors’ excess in operating costs and employees were recorded as 0.004 billion and 2862.907, respectively, which are 1.11 and 1.005 times the average value. Concurrently, the shortfalls in annual turnover and profits after tax were 0.300 and 0.383 billion, representing 1.21 and 1.07 times the average, respectively. The significant excess in employees and the shortfall in profits after tax have been identified as the primary contributors to the inefficiency within the restaurant sector. The efficiency of the entertainment facilities sector has remained consistently high from 2013 to 2022, suggesting a more effective and sustainable operation compared to other sub-sectors. The synchrony in efficiency fluctuations between the travel and restaurant sectors during the sample year, as depicted in Fig 2, indicates a certain degree of complementarity between these two sectors. The consumption of restaurant services is often intrinsically linked to travel sector, implying that enterprises may strategically expand their business and industrial chain based on this complementary relationship. Establishing a comprehensive service system, fostering a collaborative ecosystem between upstream and downstream entities, and enhancing inter-business collaboration are all strategies that could effectively enhance operational efficiency. The hotel sector’s efficiency experienced notable volatility during the sample year, with the lowest point occurring in 2020. This underscores the substantial impact of the COVID-19 pandemic on the hotel sector, a finding that aligns with the observations of Kamel et al. [31], who noted operational and financial disruptions in the hotel performance due to the pandemic. The significant decrease in room sales led to revenue losses during the pandemic. However, the non-storable nature of hotel rooms necessitated ongoing cost expenditures [65], resulting in a loss of profits after tax and operational efficiency, thereby exacerbating the financial burden on hotels.

The Mann-Whitney U test was employed to examine the disparity in efficiency between SOEs and non-SOEs. The outcomes are presented in Table 6.

**Table 6:** The efficiency and slacks of different property rights.

	SOE	Non-SOE
Efficiency	0.919	0.985
Mann-Whitney U	2444.5	
Sig	0.000	
Slacks		
Operating costs (billion)	0.098	0.277
Employees (people)	2090.271	866.973
Annual turnover (billion)	0.365	0.725
Profits after tax (billion)	0.437	0.524

The findings indicated a substantial difference in financial efficiency between various property rights systems. This outcome contradicts the research by Arocena and Oliveros [44], who reported no significant efficiency differences between SOEs and their private-owned counterparts. Upon further analysis, it was determined that the efficiency of SOEs is lower than that of non-SOEs. A notable excess in employees within SOEs was observed, with an excess of 2090.27 employees, which represents 84.73% of the average employee count within these enterprises. The primary reason for this disparity is the inability of SOEs to dismiss employees or streamline their workforce [66], which has consequently limited their efficiency.

Table 7 presents the TFP and its decomposition indices for the sample enterprises over the period from 2013 to 2022.

**Table 7:** TFP and decomposition indexes from 2013 to 2022.

Year	TFP	EC	TC
2013	0.064	0.047	0.017
2014	-0.020	-0.010	-0.010
2015	0.058	0.005	0.053
2016	0.078	0.053	0.025
2017	0.022	0.053	-0.031
2018	0.045	-0.049	0.094
2019	0.019	-0.038	0.057
2020	-0.080	0.035	-0.115
2021	0.035	0.016	0.019
2022	-0.038	0.034	-0.072
Mean	0.016	0.014	0.002

During the sample period, there was a notable progress in TFP, EC, and TC, with average annual improvements of 1.6%, 1.4%, and 0.2%, respectively. In 70% of the years analyzed, TFP values exceeded zero, suggesting that the tourism enterprises were effectively utilizing their internal resources and factors and were actively engaged in the development of new technologies and products. However, negative TFP values were observed in the years 2014, 2020, and 2022, indicating a regression in productivity during these specific years. The primary reason for this decline was the corresponding negative TC values, which suggest that a temporary pause in the introduction of advanced technology and an inability to meet market demand for products and services had a detrimental effect on TFP.

The Kolmogorov-Smirnov and Shapiro-Wilk tests have been implemented to assess the normality of the TFP, with the outcomes delineated in Table 8.

**Table 8:** The normality of TFP.

	Kolmogorov-Smirnow			Shapiro-Wilk		
	Statistic	df	Sig.	Statistic	df	Sig.
TFP	0.240	252	0.000	0.656	252	0.000

It is evident that a P-value approaching zero signifies a statistically significant departure of the TFP from a normal distribution, thus satisfying the prerequisites for employing the Mann-Whiteney U and Kruskal-Wallis tests.

The TFP of various sub-sectors is delineated in Table 9. Notably, the travel and restaurant sectors experienced progressive TFP, with average increases of 2.2% and 4.2%, respectively. EC indices across all sub-sectors indicated upward trajectories, signifying that the management levels were actively approximating industry benchmarks and effectively allocating resources. However, only the travel and restaurant sectors demonstrated TC progression; the hotel and entertainment facility sectors experienced TC regression. The TFP decline in these latter sectors was attributed to insufficient adoption of service concepts and product technologies, suggesting that TC plays a predominant role in these sub-sectors during the sample period. This outcome is rationalized by the labor-intensive nature of the hotel sector and the relatively minor impact of modern technologies on its commercial profitability [67]. The non-significant Kruskal-Wallis test result implies that, despite the TFP regression in the hotel and entertainment facility sectors, there are opportunities for improvement. It is imperative for these enterprises to invest in product research and development, refresh their service concepts, and continuously enhance their management acumen to remain competitive.

**Table 9:** The TFP and its decomposition index of sub-sectors.

	Travel	Hotel	Restaurant	Entertainment facility	Kruskal-Wallis Test	Sig
TFP	0.022	-0.008	0.042	-0.001	2.026	0.567
EC	0.014	0.013	0.032	0.004	0.585	0.900
TC	0.008	-0.020	0.010	-0.005	1.058	0.787

The TFP across varying property rights systems is delineated in Table 10. The TFP, along with its decomposition indices, for both SOEs and non-SOEs, is presented.

**Table 10:** TFP and its decomposition index of different property rights.

	SOE	Non-SOE	Mann-Whitney U	Sig
TFP	0.021	0.002	4261	0.002
EC	0.020	-0.004	5286	0.276
TC	0.001	0.005	5058	0.121

The findings reveal a marked disparity in TFP between SOEs and non-SOEs, with SOEs exhibiting a higher TFP of 0.021 compared to their non-SOE counterparts at 0.002. This divergence may be attributed to the stronger political connections of SOEs, which afford them preferential access to policy incentives, bank credit, and fiscal subsidies [15]. Consequently, SOEs are better positioned to align with industry leaders. However, no significant differences were observed between SOEs and non-SOEs in terms of EC and TC.

### 5.2. Individual level

**Fig 3:**
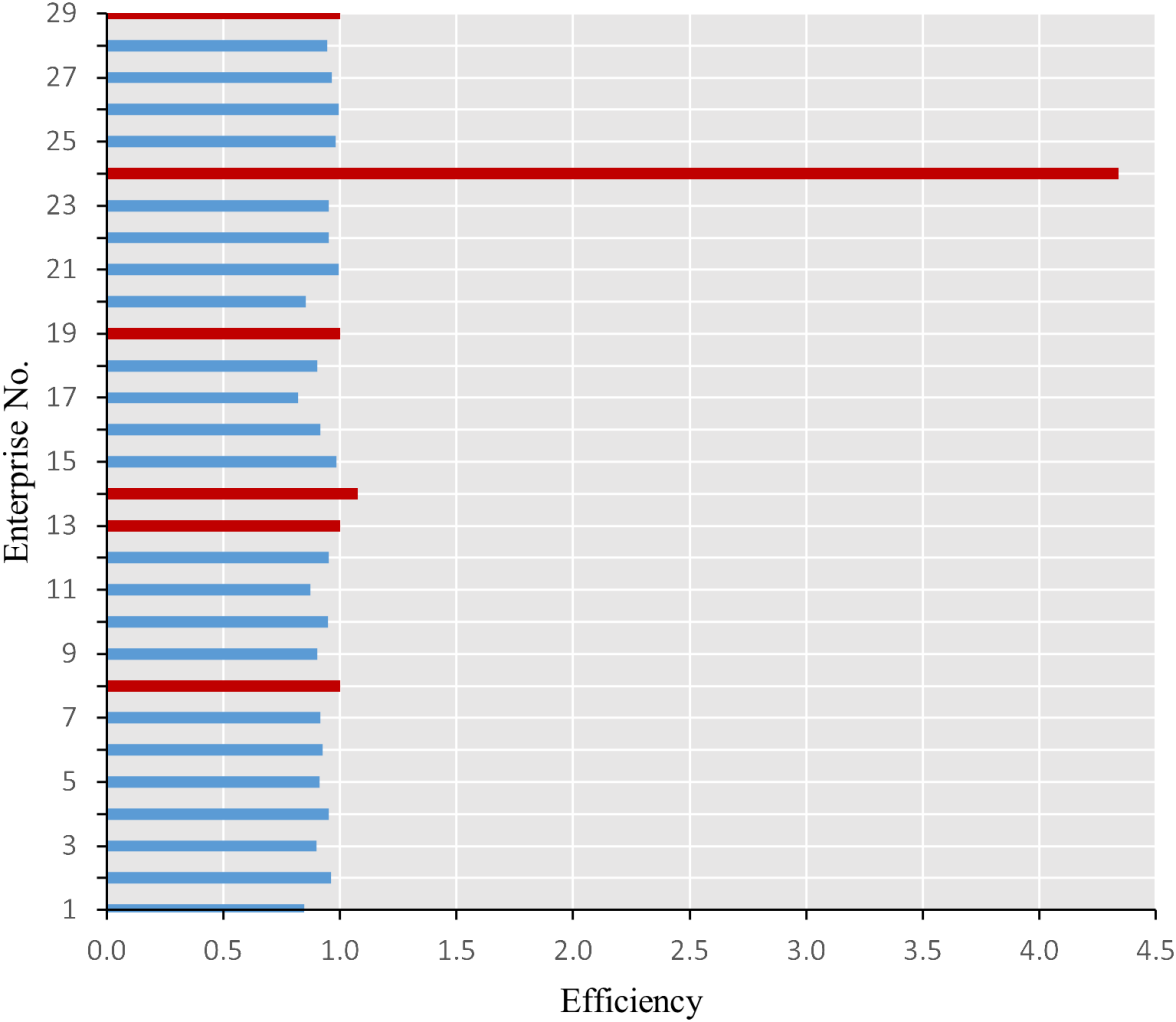
Efficiency of enterprises.

**Fig 4:**
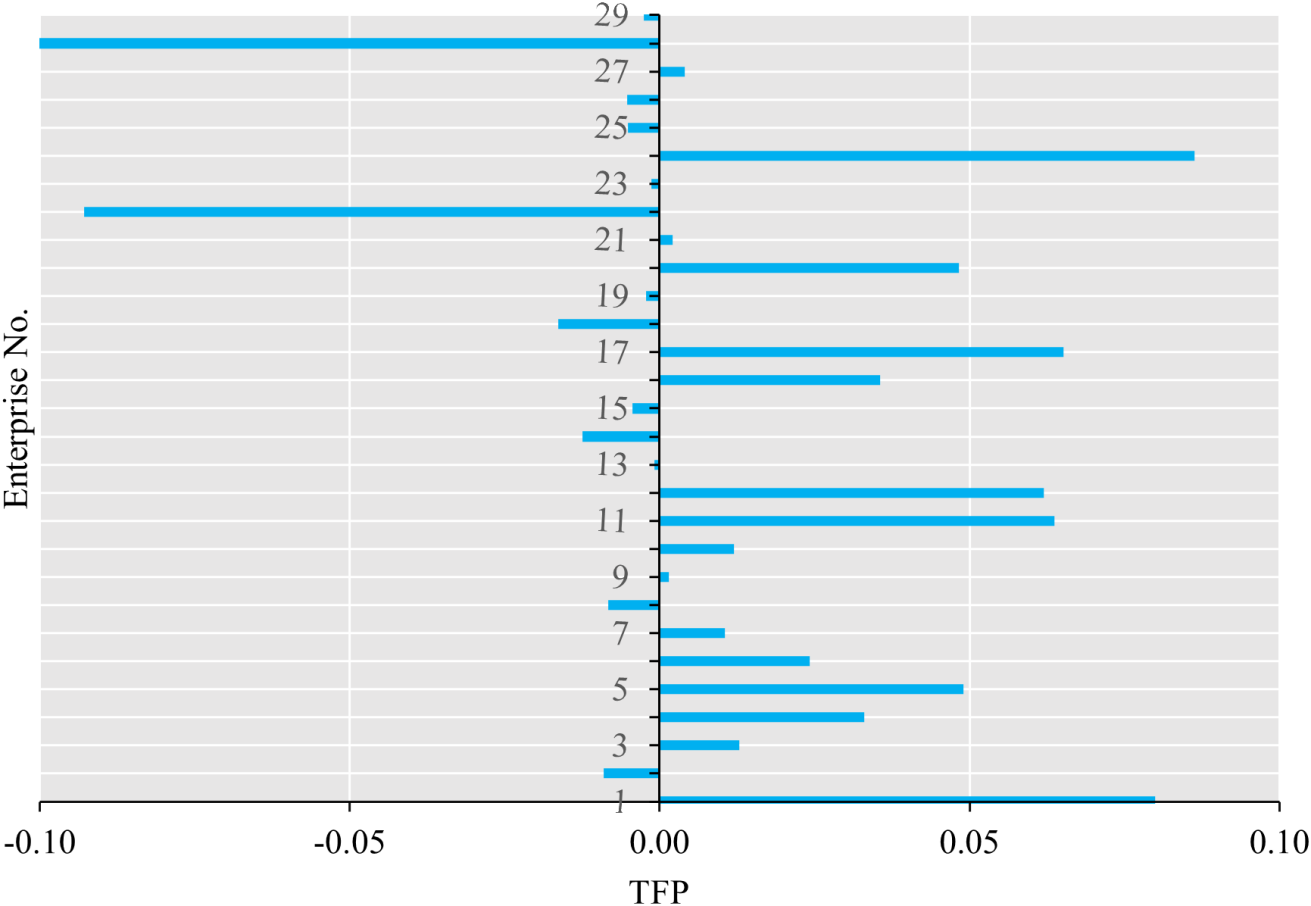
TFP of enterprises.

Figs 3 and 4 depict the efficiency and TFP, respectively, of the 29 listed tourism enterprises spanning the period from 2013 to 2022.

Fig 3 illustrates that only six enterprises effectively managed during the sample years, with a particular emphasis on enterprise No. 24, which demonstrated superior efficiency compared to its peers. This enterprise exhibited an annual efficiency score exceeding one, indicative of its consistent annual operational effectiveness. Its surplus in both annual turnover and profits after tax contributed significantly to its operational efficiency. Fig 4 presents there are sixteen enterprises experiences the TFP progression, with enterprise No. 24 again distinguishing itself with the highest TFP value. The decomposition indices reveal that TFP correlated with EC from 2013 to 2016 and with TC for the subsequent years. This suggests that TFP was predominantly influenced by EC pre-2016 and by TC post-2016. Enterprise No. 24’s EC was processed throughout most of the sample period, reflecting its exemplary management practices. This outcome can be attributed to the enterprise’s status as a flagship within China’s duty-free industry, boasting a strong reputation among significant brand suppliers, accumulating crucial brand resources, and creating heightened brand awareness in the tourism and shopping markets. Between 2013 and 2022, the enterprise continuously enhanced customer loyalty by adjusting its product structure and implementing diverse marketing activities. Concurrently, it honed its management skills through refined business management practices and the continuous introduction of talent. Regarding TC, the enterprise underwent restructuring in 2017, solidifying its position within the tourism industry chain and strengthening its main business, leading to more pronounced synergistic advantages in business operations. Additionally, enterprise No.24 established China’s sole duty-free logistics distribution system, continuously optimizing channel control and logistics distribution capabilities. The enterprise also remained committed to digital construction, advancing the application of intelligent retail scenarios in stores and executing projects such as VR/AR real-life shopping malls, thereby enhancing store customer conversion capabilities. The progress in both EC and TC facilitated the improvement of TFP.

The decision-making matrix has been constructed to evaluate the performance of listed tourism enterprises. Total assets, which represent the scale of these enterprises, average at 5.99 billion. The horizontal axis (X-axis) denotes the efficiency, the vertical axis (Y-axis) signifies the TFP, and the origin is designated as point O==. This approach diverges from previous studies that utilized average efficiency as a benchmark for matrix segmentation [46]; instead, employing a super-efficiency threshold of one provides a more scientifically rigorous criterion. The size of the bubble in the matrix correlates with the scale of an enterprise, with larger scales corresponding to greater bubble areas. Enterprises can be categorized into eight distinct types based on whether their scale exceeds the average, their efficiency surpasses one, and their TFP demonstrates progress.

**Fig 5:**
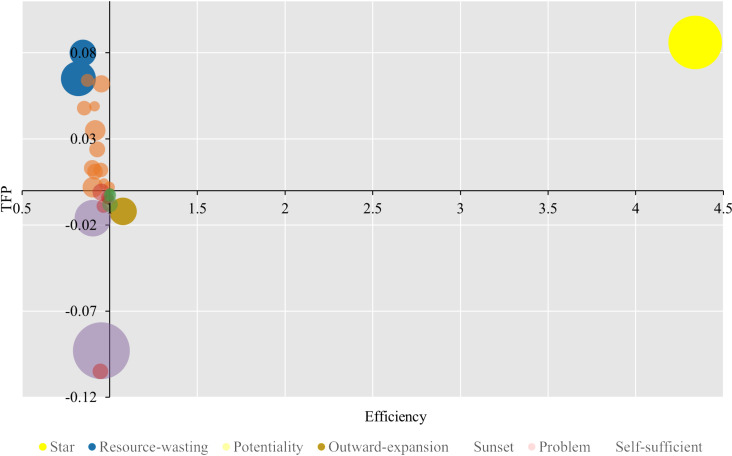
Decision-making matrix.

Fig 5 graphically represents the classification and distribution of enterprise type. The majority of enterprises are concentrated at the origin, with marginal variations in scale. A select few enterprises are situated at a considerable distance from the origin, exhibiting a larger scale. Table 11 reveals that a solitary enterprise excels in scale, efficiency, and TFP, earning the designation of a “star enterprise”. This entity serves as a benchmark for enhancing the performance of the entire industry. Among the large-scale enterprises that are not star enterprises, two are classified as resource-wasting types, and one as an outward-expansion type. These enterprises underperform in either efficiency or TFP, respectively, suggesting that scale does ensure a degree of efficiency or TFP. This phenomenon may stem from the fact that larger enterprises possess more disposable assets, enabling them to invest more substantially in research and development of new technologies. They can establish superior talent incentive systems to cultivate a more comprehensive talent structure, thereby ensuring improvements in business performance or reductions in operational costs. There are thirteen potentiality type enterprises, which, despite being small in scale and inefficient, demonstrate progress in TFP, indicating the possession of advanced technology. With planned resource allocation and moderate expansion, these enterprises may emerge as benchmarks. Six problem enterprises lag in scale, efficiency, and TFP, lacking the funds to introduce advanced productivity or the capability to allocate existing resources rationally. Enhancing the performance of these problem enterprises could significantly bolster the overall development of the industry. Two sunset enterprises, large in scale with lower efficiency and regressing TFP, challenge Arbelo et al.’s [68] conclusion that larger companies invariably possess higher efficiency and TFP due to superior product management, yielding higher quality and greater value. The remaining four self-sufficient enterprises, though small in scale, exhibit higher efficiency but regressing TFP. These self-sufficient enterprises, despite their small scale, can effectively utilize limited resources and technologies for efficient operations. None of the enterprises are classified within the small-but-excellent category, potentially due to the fact that smaller competitors frequently encounter financial constraints regarding technology investment and are unable to optimally leverage their existing resources to attain high level of both efficiency and productivity. This confirms that scale serves as a fundamental prerequisite for concurrent enhancement of efficiency and TFP, aligning with Santos et al. [12], who posit that a company’s scale is directly related to its competitiveness. Through decision-making matrix, enterprises can identify their current type by altering efficiency, TFP, or scale. That is, enterprises, except for star enterprises, can perform better by progressively moving towards the origin point in Fig 5 or by increasing in bubble size rather than becoming stars directly. This concept resonates with Cheng et al. [56], who suggest that while enterprises with higher attractiveness can serve as learning targets, the closer leader is the one that lagging enterprises should emulate. This approach is beneficial for managers to develop gradually optimized strategies rather than setting unattainable goals.

**Table 11:** Enterprise type and character.

Type	Character	Number	No.
Star	Large-size, efficient, and progress	1	24
Resource-wasting	Large-size, inefficient, and progress	2	1,17
Potentiality	Small-size, inefficient, and progress	13	3,4,5,6,7,9,10,11,12,16,20,21,27
Small-but-excellent	Small-size, efficient, and progress	0	None
Outward-expansion	Large-size, efficient, and regress	1	14
Sunset	Large-size, inefficient, and regress	2	18,22
Problem	Small-size, inefficient, and regress	6	2,15,23,25,26,28
Self-sufficient	Small-size, efficient, and regress	4	8,13,19,29

## 6. Conclusion and implications

### 6.1. Conclusion

The tourism sector has experienced substantial growth and transformation in recent years [69]. Existing researches indicate that a conducive environment can markedly enhance stress relief and emotional well-being [70,71], which has in turn driven the growing demand among consumers for high-quality travel experiences. In an effort to more effectively satisfy consumer demands and enhance the developmental quality of the tourism industry, this study conducts a performance analysis of Chinese tourism enterprises. Utilizing DEA and the Luenberger index to assess the efficiency and TFP of the enterprises, the findings indicate that the tourism industry exhibits a relatively high level of overall efficiency, along with notable progress in TFP and its decomposition indices. Nonparametric tests are employed to discern differences in efficiency and TFP among various sub-sectors and property rights systems. The analysis reveals disparities in efficiency and TFP across these sectors and systems; notably, the entertainment facilities sector demonstrates the highest efficiency, in contrast to the restaurant sector, which shows significantly lower efficiency. Additionally, the efficiency of SOEs is found to be significantly lower than that of non-SOEs. Regarding TFP, while differences among sub-sectors are not pronounced, a significant difference in TFP is observed between SOEs and non-SOEs, although the decomposition of TFP does not vary substantially between these two types. The tourism enterprises are plotted within a decision-making matrix according to their efficiency, TFP, and scale, enabling each enterprise to identify its type and to formulate competitive strategies accordingly.

### 6.2. Implications

The present study yields theoretical and practical implications for the tourism industry.

Theoretically, this research pioneers the application of the negative DEA model to ascertain the efficiency of tourism enterprises, addressing the previous research’s limitation of employing transformed data for negative indicators, which could introduce bias in the results. Furthermore, the TFP of these enterprises is measured using the Luenberger index, which is capable of accounting for input reduction and output expansion simultaneously, without necessitating proportional changes in each variable. This research contributes to the theoretical framework by evaluating the performance, encompassing both efficiency and TFP, of the tourism industry as a whole, rather than focusing on a single sub-sector. To the best of our knowledge, this is the inaugural study to employ nonparametric statistical analysis to discern performance disparities attributable to property rights systems within the tourism industry, thereby expanding the investigative approaches in evaluating the performance of tourism enterprises. Additionally, the decision-making matrix aids tourism enterprises in devising appropriate strategies by considering a holistic view of performance. Our study elucidates the disparities in the operational performance of tourism enterprises attributable to the characteristics of property rights system. Notably, these discrepancies are not uniform across all metrics. SOEs exhibit superior TFP, yet their efficiency does not align with this advantage. This finding, to some extent, augments the extant theory regarding the impact of property rights systems [72], suggesting that property rights systems do not unilaterally determine corporate performance.

Practically, our research offers valuable insights to managers of tourism enterprises. Given the tourism industry’s susceptibility to economic fluctuations, it is advisable for managers to conduct a thorough review of their operational environments. It is recommended that they focus on reducing operating costs and employees while concurrently striving to increase annual turnover and profits after tax. Notably, the restaurant is grappling with the most pronounced challenges in terms of excessive operating costs and employees, as well as a scarcity of annual turnover and profits after tax. Therefore, managers in this sector must pay closer attention to the dynamic of inputs and outputs relative to their peers in other sub-sectors. Moreover, our study discloses a pattern of synchronized efficiency fluctuations within certain sub-sectors, namely the travel and restaurant sectors. This suggests that the extension of the tourism industry’s value chain could markedly improve the operational performance of tourism enterprises. For instance, forging strategic alliances, partnerships, and collaborative initiatives across diverse sub-sectors within the tourism industry can lead to synergistic benefits. Additionally, it is recommended that SOEs embrace market-oriented management practices to bolster operational efficiency. The establishment of a market-oriented performance evaluation system is essential, which should enable the selection and hiring of managerial talent through market-driven approaches and motivate both management and staff to enhance work efficiency and innovation capabilities. Lastly, it is emphasized that managers of tourism enterprises should acknowledge the unique competitive position of their enterprises within the industry. Crafting management strategies based on this positioning can significantly enhance an enterprise’s competitiveness. Specifically, tourism enterprises may opt for different priorities in performance improvement based on their specific types. Some may need to allocate resources judiciously to improve efficiency, others may focus on adopting innovative technologies to increase TFP, and the rest may seek to expand their scale. Naturally, when internal and external conditions permit, multiple adjustments can be made concurrently.

## Supporting information

S1 Original Data. The original data are available from the database of CSMAR from 2013 to 2022 to replicate our findings in this article.
(ZIP)
